# Identifying key risk factors for Chinese international students: a hybrid AHP–DEMATEL–cross-reinforcement matrix approach with policy implications

**DOI:** 10.3389/fsoc.2025.1610036

**Published:** 2025-07-10

**Authors:** Hongwei Li, Lei Wang, Junhong Gao

**Affiliations:** College of Economics and Management, Shandong University of Science and Technology, Qingdao, China

**Keywords:** Chinese student studying abroad, key risk factors, AHP, DEMATEL, cross-reinforcement matrix

## Abstract

Chinese students usually face risks from various aspects in the process of studying abroad. The use of the analytic hierarchy process alone ignores the interplay between the influencing factors and lacks systematic thinking about the identification of key influencing factors due to the intricacies of the factors affecting and constraining these risks. Therefore, we utilize the DEMATEL method and cross-reinforcement matrix to improve the weights obtained from AHP and to enhance the accuracy and scientific rigor of the weight vectors. Finally, five factors with the largest weights of risk factors affecting international students are identified through the analysis. They are self-management ability, language ability, policy of the host country, economic conditions of the host country, and values. Appropriate risk response countermeasures are proposed to reduce the risk potential of international students based on the results. Exploring the risk factors affecting students studying abroad can provide a reference for Chinese students to predict and control the risks of studying abroad. It can also provide support for international institutions to recruit, manage, and assist Chinese students.

## 1 Introduction

China's education system is constantly reforming and changing with the development of globalization since the reform and opening-up policy was introduced. During the last 40 years, studying abroad is becoming more and more popular, the number of Chinese students studying abroad have increased greatly, and the destination countries are becoming more diverse (Center for China and Globalization, 2024). This has formed a special migration group of Chinese students studying abroad. However, some students lack sufficient knowledge and understanding of the risks or potential problems associated with studying abroad, which has accompanied the rapid growth in the number of Chinese students studying abroad. They also lack sufficient knowledge of their own abilities and guidance and advice in the process of studying abroad. All of these factors can gradually change their experience of studying abroad from initial excitement to later pressure, and potentially even to failure (Mehar Singh, 2016; Alemu and Cordier, 2017; Kobayashi, 2022). According to EIC Education's statistic, over 80% Chinese students with undergraduate degree or above continue learning through studying abroad (EIC Education, 2023), making up the majority of the overseas student population. This group faces unique challenges. For instance, postgraduate students need to deal with academic accreditation and the pressure of interdisciplinary research, while undergraduates are more concerned about cross-cultural adaptation and their ability to live independently. This study focuses on Chinese students with a bachelor's degree or above who study abroad, covering major destinations such as the United States, the United Kingdom, Australia, and Canada, and analyzes the key risk factors they encounter during their overseas study. Recent surveys indicate that 28% of Chinese students report moderate-to-severe academic stress, while 15% experience culture shock leading to academic delays (Wang et al., 2023). For instance, study revealed that self-management (Julia et al., 2022) and language barriers (Abdullah Ahmed, 2023) are important factors. These statistics underscore the critical need to systematically identify risk factors and inform pre-departure preparation. Thus, these factors will affect the learning and living experience and quality during studying abroad; they also expose the education quality of Chinese students studying abroad to the risk of uncertainty. While previous studies (Li et al., 2022; Wang et al., 2023) have analyzed individual risks like language barriers or policy changes, they often overlook the interdependencies between factors, such as how host country economic conditions interact with students' self-management abilities. Post-2020 challenges, including COVID-19-related mobility restrictions and rising geopolitical tensions (Sina, 2021; Zhao and Xue, 2023), further highlight the need for a holistic framework that captures both direct and indirect risk pathways.

We first categorized and summarized the risks that Chinese students may face in the process of studying abroad according to the research of scholars and discussions within the research group. We constructed a risk influencing factor framework for international students containing 22 indicators. The 22 risk indicators were developed by synthesizing four major risk categories identified in prior literature: circumstance risks (CR, five indicators, e.g., host country policy), language and cultural risks (LC, five indicators, e.g., cross-cultural adaptation), academic risks (AR, seven indicators, e.g., self-management ability), and opportunity risks (OR, five indicators, e.g., psychological pressure). This framework aligns with studies by Bahna (2018) and López et al. (2016), which similarly categorized study-abroad risks into environmental, cultural, academic, and socioeconomic dimensions. Secondly, the established indicator system was evaluated, and the weights of the evaluation indicators obtained by AHP were corrected by using the DEMATEL method and cross-reinforcement matrix method. The analysis results take into account both the interaction between various risk factors and their relative importance, making the evaluation of risk indicator weights more accurate and scientific. Finally, the important indicators affecting the risk factors of international students are derived, and corresponding coping countermeasures are proposed based on the results to help international students navigate their study abroad experience more effectively. This paper addresses the limitation that previous studies have only explored the influencing factors of international students from a single perspective. This study innovates by integrating the analytic hierarchy process (AHP) with the Decision-Making Trial and Evaluation Laboratory (DEMATEL) and cross-reinforcement matrix, a hybrid approach that simultaneously accounts for factor weights and interdependencies. Unlike single-method studies, this framework quantifies both direct and indirect risk pathways, providing a more systematic risk assessment for Chinese students and institutions. It offers a reference for students to plan, prepare, and participate in study abroad.

## 2 Materials and methods

### 2.1 Literature review

There has been an abundance of articles exploring the factors influencing international students, covering all levels. However, the research on the influencing factors of students studying abroad has various focuses and results. Politics (Bratsberg, 1995), economy (Bahna, 2018; López et al., 2016), systems (Gopalan et al., 2019), education (Roshid and Ibna Seraj, 2023), and social culture (Park, 2019; Sezer et al., 2021) can have a great impact on international students.

Drawing from the stress-coping theory and complex systems theory, this review categorizes risks into circumstances, cultural, academic, and opportunity domains, examining how individual and contextual factors interact to shape study-abroad outcomes.

Circumstances risks mainly focus on the direct impact of the host country's political, policy, legal, security, and economic macro-environment on the adaptation of international students. Students will relate and respond to various ideologies through negotiation, development, and reconstruction of their ideologies and positions during the process of their studies abroad (Choi, 2021). Ideology is concerned with racialism (Park and Choi, 2022). Teachers' ideologies impact their teaching approaches and attitudes toward the students' heritage language and culture (Gu et al., 2019). Sung found that ideology has an impact on the acceptance of using a lingua franca (Sung, 2020), such as English. Local students prefer monolingual ideologies. However, another study found that foreign students prefer a lingua franca, even if it is not English (Kobayashi, 2022). As Sung pointed out, the perceived resistance against the use of a lingua franca by local students means that the language norms operating in the university cannot be pre-determined. The contradictory language ideologies concerning monolingualism/multilingualism coexist in international universities (Sung, 2020). When Chinese students study in non-English-speaking countries where English is used as a lingua franca of the university, they bear the risk of language uncertainty due to ideology. This risk brings difficulties for them in learning, communicating with each other, and integrating into the university community. Public policy shifts in immigration and education have a clear influence on Chinese students studying abroad (Kobayashi, 2022). Competing perceptions of international students as threatening or beneficial lead to a policy wave. They are considered by students studying abroad as factors such as political stability, a safe environment in the country, a multicultural society, a low cost of living, and simplified immigration procedures (Mehar Singh, 2016). Thus, students studying abroad bear the risk of policy changes that may result in visa or resident permit interruptions. Law is the embodiment of ideology and national policy (Maslennikova, 2021), which reflects whether the investment in studying abroad is worthwhile in the long run (Lam et al., 2017). Changes in policies and laws related to international students, such as immigration, visa, and other policies and regulations, will affect the development planning of students studying abroad (Graham and Pottie-Sherman, 2022). Moreover, changes in visa policy will lead to interruptions in studying abroad (Sina, 2021). Some scholars believe that policy challenges have the greatest impact on international students through interview analysis (Zhao and Xue, 2023). In addition, international students are considered a disaster-vulnerable group after many horrible murders occurred involving students studying abroad (Ryoo and Cheung, 2021). Therefore, adequate attention should be paid to the safety of international students (Yu et al., 2021).

Language and cultural risks mainly revolve around language barriers, cross-cultural interaction, value differences, and independent adaptation abilities, among other cultural-level challenges. Language competence is a primary issue for Chinese students in intercultural communication (Gu, 2018). The limitation of language ability is manifested in many aspects; for example, language skills have a bearing on future career development (Ren et al., 2023). Language proficiency correlates with academic performance (Wang et al., 2023), which may result in learning difficulties or even an inability to complete their studies. Language skills also affect communication with others (Mitchell and Güvendir, 2023). Discomfort during cultural contact will affect the quality of life and academic achievements of individuals (Popescu and Buzoianu, 2017). Students come from different cultural backgrounds during the process of cross-cultural adaptation (Dahal et al., 2018). This means they have different understandings of values, lifestyles, religions, and beliefs. They may encounter risks due to cultural differences (Byrne et al., 2019). Students have to face cultural re-adaptation when they return to China after graduating from abroad universities (Kim, 1977). The ability of study abroad students to deal with problems independently is related to their social adaptation and career ambition after graduating from abroad universities (Yu, 2021).

Academic risks mainly cover factors directly affecting academic performance, such as information access, major selection, teaching management, self-management, and academic credential recognition. Academic adjustment positively affects psychological and sociocultural adjustment (Sheng et al., 2022). However, inappropriate academic choices can hurt international students (Yu et al., 2023). Education agencies are used by most Chinese students studying abroad in various processes of studying abroad (Pimpa, 2003). However, they may also face information asymmetries that can affect their judgment (Nikula and Kivistö, 2018). There is a significant difference in the quality of education between international and domestic students (Lee et al., 2019). It has been argued that fragmented learning of both language and competencies can lead to risks in instructional management (Sun, 2022). Alemu and Cordier found that academic and educational quality (Alemu and Cordier, 2017), as well as life and support experiences, will affect student satisfaction. Certification of qualifications earned abroad is only valid if they are recognized by the Chinese Ministry of Education. It is a comprehensive assessment of the learning process of study abroad students (Chinese Service Center for Scholarly Exchange, 2018) as well as a certification of learning ability. Growing up in a comfortable environment in China, Chinese students lack self-management and self-care abilities in life. They have less stress tolerance (Cai, 2017), which may become a potential risk when studying abroad. Some international students also experience pressure related to tuition fees, which not only affects the number of international applicants for relevant majors (Elliott and Soo, 2013) but also impacts whether students complete their studies (Bradley and Migali, 2019).

Opportunity risks mainly concern uncertainties related to long-term development, including career development, economic pressure, mental health, social integration, and sudden security incidents. Feng explained the impact of educational gaps and career development on Chinese students studying abroad (Feng, 2018). International students are more likely to come from families with a high level of economic status, which can easily cause financial pressure on some families with average economic conditions (Bahna, 2018). International students have less access to public resources due to financial, informational, language, or cultural difficulties (Song et al., 2020). Studies have found that mental health problems among students abroad have long been widespread (Wang H. et al., 2022; Wang Y. et al., 2022) Chinese students are far from home and parents, living and studying overseas alone during the growth period of studying abroad. They are in a completely unfamiliar cultural environment, which can easily produce homesickness and loneliness (Mekonen and Adarkwah, 2023; Sezer et al., 2021). After being abroad for a long time, Chinese students have to go through cultural re-adaptation once they return to China (Kim, 1977). They will encounter a series of social difficulties (Chen et al., 2022). In addition, accidents are also an important factor to consider when studying as an international student (Love et al., 2023).

The influencing factors of the risks faced by Chinese students studying abroad can be categorized as circumstances risk, language and cultural risk, academic risk, opportunity risk, etc. Only considering the importance among the risk factors or the mutual influence of the risk factors will lead to an incomplete understanding of the risk-influencing factors due to the complex relationships between them. Therefore, we adopt DEMATEL and a cross-reinforcement matrix to improve the index weights obtained by AHP to identify the key risk factors affecting international students. Unlike prior single-method studies (Zhao and Xue, 2023), this research employs a hybrid AHP–DEMATEL model to quantify both factor importance and interdependencies, providing a more nuanced understanding of risk pathways. AHP was selected for its capability to handle hierarchical multi-criteria decision-making, while DEMATEL excels at modeling causal relationships in complex systems. Compared to alternatives like Fuzzy TOPSIS, this combination uniquely addresses the “interplay neglect” issue in prior risk studies. We consider both the importance of risk factors and the degree of importance of risk factors, making the evaluation results more accurate. Section 2.2 details the risk factor classification process, while Section 2.3 explains the hybrid methodology used to prioritize and model these factors.

### 2.2 Selection and determination of risk factors of studying abroad

This study defines “risks” as challenges that significantly impact Chinese students' academic performance, mental wellbeing, or cultural adaptation during their study-abroad period. The influencing factors analyzed are those that directly or indirectly contribute to these risks. The risks of Chinese students studying abroad are classified into four clusters according to the source of risk based on the views and perspectives of the aforementioned scholars. The four clusters are set as the first-level indicators, including circumstance risks, language and cultural risks, academic risks, and opportunity risks. The 22 secondary indicators were derived through a three-step process: (1) systematic literature review identifying recurring risk themes; (2) focus groups with returned students to validate contextual relevance; (3) expert panel consensus (Delphi method) to refine categories. A total of 22 factors were determined as secondary indicators and sorted into the above four clusters. The risks of Chinese students studying abroad are taken as an integrative indicator. Thus, the indicator system is established.

The index system has been discussed and compared many times to avoid overlapping or omission of risk-influencing factors. The determined risks of overseas students and the corresponding influencing factors are listed in Table 1.

**Table 1:** Influencing factor index system of Chinese students studying abroad.

**First-level indicators**	**Secondary indicators**	**Code**	**Indicator provenance**
Circumstances risk (CR)	Ideology of host country	CR1	Sung, 2020; Choi, 2021; Park and Choi, 2022; Mitchell and Güvendir, 2023
	Policy of host country	CR2	Mehar Singh, 2016; Sina, 2021; Kobayashi, 2022; Zhao and Xue, 2023
	Differences in laws and regulations	CR3	Lam et al., 2017; Graham and Pottie-Sherman, 2022
	Public security environment of host country	CR4	Mehar Singh, 2016; Ryoo and Cheung, 2021; Yu et al., 2021
	Economic conditions of host country	CR5	López et al., 2016; Bahna, 2018
Language and cultural risk (LC)	Language ability	LC1	Gu, 2018; Mitchell and Güvendir, 2023; Ren et al., 2023; Wang et al., 2023
	Cross-cultural adaptation	LC2	Popescu and Buzoianu, 2017; Dahal et al., 2018
	Values	LC3	Byrne et al., 2019
	Cultural re-adaptation of students return to China	LC4	Kim, 1977
	Ability to deal with problems independently	LC5	Yu, 2021
Academics risk (AR)	Information acquisition	AR1	Pimpa, 2003; Nikula and Kivistö, 2018
	Academic choice	AR2	Sheng et al., 2022; Yu et al., 2023
	Teaching management	AR3	Sun, 2022
	Education quality	AR4	Alemu and Cordier, 2017; Lee et al., 2019
	Academic certification authentication	AR5	Chinese Service Center for Scholarly Exchange, 2018
	Self-management ability	AR6	Cai, 2017
	Study expenses	AR7	Bradley and Migali, 2019; Mehar Singh, 2016
Opportunity risk (OR)	Work and future development	OR1	Feng, 2018
	Economic pressure due to studying abroad	OR2	Bahna, 2018; Mehar Singh, 2016
	Psychological pressure	OR3	Mekonen and Adarkwah, 2023; Popescu and Buzoianu, 2017; Sezer et al., 2021; Wang H. et al., 2022; Wang Y. et al., 2022
	Social dilemma	OR4	Chen et al., 2022; Song et al., 2020
	Accident	OR5	Love et al., 2023

### 2.3 Method

There are numerous studies on study-abroad risk, which have been elaborated on in the previous articles. It can be found that scholars' research mainly focuses on the selection of risk factors for students studying abroad, and the indicators of the constituency are further analyzed. Few scholars have synthesized the indicators at various levels and considered the complex relationship between the risk factors. For example, “language ability (LC1)” has an extremely strong impact on “cross-cultural adaptation (LC2)” (Gu, 2018). “Cross-cultural adaptation (LC2)” has a strong impact on “work and future development (OR1)” and “psychological pressure (OR3)” (Dahal et al., 2018; Kong et al., 2023; Wang H. et al., 2022; Wang Y. et al., 2022). Furthermore, “work and future development (OR1)” will have an impact on “language ability (LC1)” (Gai et al., 2022). These complex influencing relationships also make it difficult to identify the key influencing factors related to the risk of Chinese students studying abroad. Determining the specific assessment methods for key factors requires obtaining relatively high-quality data in advance during the analysis process. Effective data collection for large groups is difficult in practice and may have limitations such as distorted data collection because international students are relatively dispersed. Therefore, the lack of accurate and high-quality data makes it difficult to determine the importance of the influencing factors. These are important issues that need to be addressed in the assessment of risk factors for international students. We used the Delphi method and AHP method to determine the importance of each risk to better carry out risk assessment in the absence of data. The influence relationship between risk factors can lead to unstable evaluation results. We utilized the DEMATEL method as well as the cross-reinforcement matrix method to correct the weights of the evaluation indexes computed by the AHP method, making the results of the assessment more credible and accurate.

The Delphi method is also known as the expert opinion method or expert survey method. It is a group decision-making method for a specific issue, consulting experts in related fields in writing, relying on the experience, knowledge, and comprehensive analysis ability of the experts, collecting and summarizing the opinions of the experts on the issue over and over again. Then it adopts mathematical and statistical methods to collate their opinions and ultimately obtains a more consistent result from the experts (Rader, 1950). We determine the relative importance and degree of influence of each risk factor based on the knowledge and experience of the experts. We defined a 0–9 level judgment matrix scale to evaluate the relative importance of the risk factors (Sumo et al., 2023) and a 0–4 scale to assess the relationship between the selected 22 factors. In the influence relationship scale, 0 = no influence, 1 = weak influence, 2 = strong influence, and 4 = extremely strong influence (Annika et al., 2025). Twenty-two factors in the form of a scale were sent to 17 experts to assess the importance and impact of each pair of risk factors. These experts included academics with experience in overseas study, international education researchers, teachers organizing overseas study, officials of overseas study regulatory bodies, and senior managers of overseas study agencies. Seventeen experts were purposefully selected to ensure multi-stakeholder representation: five academic researchers (with ≥10 years of study-abroad risk publications), four international education administrators, three policy officials (from China's Ministry of Education), and five industry professionals (overseeing >1,000 study-abroad cases annually). This mix ensures both theoretical rigor and practical relevance, aligning with Delphi method guidelines for complex problem-solving (Rader, 1950). The degree of influence of each factor was taken from the evaluation results of more than half of the 17 experts who shared the same opinion. Three rounds of expert surveys were conducted over 8 weeks. Consensus was assessed using the Kendall coefficient of concordance (W), which reached 0.78 (*p* < 0.001) after Round 3, indicating high agreement. This exceeds the acceptable threshold of 0.7.

AHP, first proposed by Saaty in the 1970s, is a frequently used evaluation and decision-making method in system engineering. It is suitable for dealing with multi-objective and multi-level complex system problems. It can express and deal with people's subjective judgments in quantitative form. It is a subjective analysis method combining qualitative and quantitative aspects (Saaty, 1987). The consistency of each judgment matrix is tested according to the nine-level scale of the judgment matrix. The sum-product method is used for the calculation of indicator weights and consistency tests to ensure the credibility of hierarchical single ordering.

DEMATEL was used to construct a comprehensive influence matrix, quantifying direct and indirect effects between the 22 factors. The cross-reinforcement matrix then adjusted AHP weights by incorporating factor interdependencies, addressing the limitation of AHP's assumption of factor independence. This hybrid approach enhances weight accuracy.

Step 1: normalize the elements of each column of the judgment matrix.


(1) $$\begin{array}{l} b_{ij}=r_{ij}/\sum_{i=1}^{n}r_{ij}\left(i=1,2,...n;j=1,2,...n\right) \end{array}$$

Step 2: the normalized judgment matrices are summed by rows.


(2) $$\begin{array}{l} c_{i}=\sum_{j=1}^{n}b_{ij}\left(i=1,2,...n\right) \end{array}$$

Step 3: normalize the vector.


(3) $$\begin{array}{l} w_{i,}=c_{i}/\sum_{j=1}^{n}c_{i}\left(i=1,2,...n\right) \end{array}$$

Then $W={\left[w_{1,}w_{2},...,w_{n}\right]}^{T}$ is the desired weight vector.

Step 4: calculate the maximum eigenvalue of the judgment matrix.


(4) $$\begin{array}{l} \lambda_{\max}=\frac{1}{n}\sum_{i=1}^{n}\frac{{\left(Rw\right)}_{i}}{w_{i}} \end{array}$$

Step 5: conduct a consistency check.


(5) $$\begin{array}{l} CI=\frac{\lambda_{\max}-n}{\text{n}-1};CRM=\frac{CI}{RI} \end{array}$$

where λ_max_ is the largest characteristic root of the judgment matrix, and n is the order of the judgment matrix. *RI* is the average random consistency index, and the value of RI can usually be obtained by checking the table. The result of hierarchical single sorting is considered satisfactory only when the value of *CRM* is < 0.1. Otherwise, the judgment matrix needs to be adjusted until it meets the requirements.

## 3 Result

The judgment matrices for each first-level indicator and secondary indicator were obtained by the Delphi method as shown in Tables 2, 3.

**Table 2:** The judgment matrix of the first-level indicators.

**Indicator**	**CR**	**LC**	**AR**	**OR**
CR	1	2	1	3
LC	1/2	1	1/3	1
AR	1	3	1	3
OR	1/3	1	1/3	1

**Table 3:** The judgment matrices of secondary indicators.

**Indicator**		**CR1**	**CR2**	**CR3**	**CR4**	**CR5**	**Indicator**		**LC1**	**LC2**	**LC3**	**LC4**	**LC5**
CR1		1	1/4	3	1	1/2	LC1		1	7	1	3	5
CR2		4	1	5	3	2	LC2		1/7	1	1/5	1/3	1/2
CR3		1/3	0.2	1	1/3	1/5	LC3		1	5	1	3	4
CR4		1	1/3	3	1	1/3	LC4		1/3	3	1/3	1	2
CR5		2	1/2	5	3	1	LC5		1/5	2	1/4	1/2	1
**Indicator**	**AR1**	**AR2**	**AR3**	**AR4**	**AR5**	**AR6**	**AR7**	**Indicator**	**OR1**	**OR2**	**OR3**	**OR4**	**OR5**
AR1	1	3	1	1	1	1/5	1/3	OR1	1	3	5	1/3	1/5
AR2	1/3	1	1/3	1/3	1/3	1/7	1/4	OR2	1/3	1	3	1/4	1/6
AR3	1	3	1	1	1	1/4	1/2	OR3	1/5	1/3	1	1/5	1/7
AR4	1	3	1	1	1	1/4	1/2	OR4	3	4	5	1	1/4
AR5	1	3	1	1	1	1/4	1/2	OR5	5	6	7	4	1
AR6	5	7	4	4	4	1	3						
AR7	3	4	2	2	2	1/3	1						

We have found the combined weights of the risk-influencing factors as a result of the above analysis, as shown in Table 4.

**Table 4:** Indicator weights obtained by AHP.

**First-level indicators**	**Secondary indicators**	**Weights**	**Maximum characteristic root**	***CI* Value**	***CRM* value**	**Combined weights**
Circumstances risk (CR)/0.3472	CR1	0.1300	5.1090	0.0270	0.0250	0.0451
	CR2	0.4136				0.1436
	CR3	0.0552				0.0192
	CR4	0.1290				0.0448
	CR5	0.2722				0.0945
Language and cultural risk (LC)/0.1423	LC1	0.3810	5.0480	0.0120	0.0110	0.0542
	LC2	0.0527				0.0075
	LC3	0.3427				0.0488
	LC4	0.1397				0.0199
	LC5	0.0839				0.0119
Academics risk (AR)/0.3829	AR1	0.0905	7.0910	0.0150	0.0110	0.0347
	AR2	0.0379				0.0145
	AR3	0.0974				0.0373
	AR4	0.0974				0.0373
	AR5	0.0974				0.0373
	AR6	0.3948				0.1512
	AR7	0.1846				0.0707
Opportunity risk (OR)/0.1276	OR1	0.1447	5.3760	0.0940	0.0850	0.0185
	OR2	0.0771				0.0098
	OR3	0.0415				0.0053
	OR4	0.2294				0.0293
	OR5	0.5073				0.0647

Full weights for all 22 secondary indicators are reported in Table 4, with self-management ability (AR6, 0.1512) and language ability (LC1, 0.0542) ranking highest in the academic and language/cultural risk categories, respectively. Host country policy (CR2, 0.1436) and economic conditions (CR5, 0.0945) emerged as key environmental factors, while values (LC3, 0.0488) ranked fifth overall.

The AHP results are corrected with the help of the cross-reinforcement matrix and DEMATEL method due to the mutual influence of the four criterion-level indicators.

The DEMATEL method was utilized to calculate a comprehensive impact matrix.

Step 1: determine the relationship between the influencing factors. This has been specified earlier in the Delphi method section. The expert opinions were summarized to obtain a matrix of direct influence relationships for all risk factors.

Step 2: initialize the direct influence matrix *G* = [_*g*_*ij*_]*n*×*n*_. Construct the direct influence matrix based on the expert evaluation results. *g*_*ij*_ is the direct influence degree of factor *i* on factor *j*, which reflects the direct influence relationship between the factors.

Step 3: standardize the direct influence matrix. Let *S* = [_*s*_*ij*_]*n*×*n*_ be the standardized influence matrix. The sum of each row of the matrix *G* is calculated, and the maximum one is denoted by MAX.


(6) $$\begin{array}{l} S={\left[g_{ij}/MAX\right]}_{n\times n} \end{array}$$

Step 4: calculate the integrative influence matrix *T*. The unit matrix is denoted by *I*, and the *T* value is calculated to examine the integrative influence relationship, including the indirect influence relationship:


(7) $$\begin{array}{l} T={\left[t_{ij}\right]}_{n\times n}=\underset{l\to \infty }{\lim}\left(S+S^{2}+\ldots +S^{l}\right)=S{\left(I-S\right)}^{-1} \end{array}$$

The direct impact matrix obtained through the Delphi method is shown in Table 5.

**Table 5:** Direct impact matrix.

**Indicator**	**CR1**	**CR2**	**CR3**	**CR4**	**CR5**	**LC1**	**LC2**	**LC3**	**LC4**	**LC5**	**AR1**	**AR2**	**AR3**	**AR4**	**AR5**	**AR6**	**AR7**	**OR1**	**OR2**	**OR3**	**OR4**	**OR5**
CR1	0	2	2	1	2	0	1	1	1	0	1	0	1	1	0	0	0	1	0	1	1	1
CR2	1	0	1	2	2	0	1	1	1	0	1	1	1	1	1	0	1	2	1	1	0	1
CR3	0	0	0	1	0	0	1	1	1	0	0	2	1	1	1	0	0	1	0	0	0	1
CR4	0	1	0	0	1	0	1	1	1	0	0	1	1	1	0	0	0	1	0	1	0	1
CR5	1	1	0	2	0	1	1	1	1	0	0	1	1	1	0	0	1	1	1	0	0	0
LC1	0	0	0	0	0	0	4	1	1	1	4	4	1	4	0	1	4	4	1	2	2	1
LC2	0	0	0	0	0	1	0	1	2	2	2	0	1	2	0	1	0	2	0	2	2	0
LC3	0	0	0	0	0	2	2	0	2	1	1	2	2	1	0	2	1	2	1	2	1	0
LC4	0	0	0	0	0	0	1	1	0	1	1	1	0	0	0	0	0	2	2	1	1	0
LC5	0	0	0	0	0	0	1	0	2	0	2	1	0	1	0	2	0	2	1	2	1	2
AR1	0	0	0	0	0	0	1	0	2	1	0	2	1	2	1	0	1	2	1	1	1	1
AR2	0	0	1	0	0	1	1	1	2	0	0	0	0	2	1	0	2	2	2	1	0	0
AR3	0	0	0	0	0	2	0	1	0	0	0	0	0	4	2	0	1	1	1	1	0	0
AR4	0	0	0	0	0	2	0	2	0	0	0	1	1	0	4	0	1	2	0	1	0	0
AR5	0	0	0	0	0	0	0	0	1	0	0	2	1	1	0	0	0	2	2	1	1	0
AR6	0	0	0	0	0	2	2	1	2	1	1	1	0	1	1	0	1	2	1	1	1	1
AR7	0	0	0	0	0	0	0	0	0	0	0	1	0	0	0	0	0	0	1	1	0	0
OR1	0	0	0	0	0	2	1	1	1	0	0	1	0	0	0	0	0	0	2	1	1	0
OR2	0	0	0	0	0	0	1	2	0	0	0	1	0	0	0	0	0	0	0	1	1	0
OR3	0	0	0	0	0	0	1	1	0	2	2	2	0	0	0	0	0	0	0	0	1	0
OR4	0	0	0	0	0	1	1	0	2	0	2	1	0	0	0	0	0	2	0	2	0	1
OR5	0	0	0	0	0	0	0	0	0	0	0	1	1	0	1	0	1	1	1	1	0	0

Then, the cross-reinforcement matrix is used to correct weight vectors.

Step 1: the weight value of each evaluation index obtained by AHP is *w*_*i*_ after normalization. It is called the value of the weights.

Step 2: determine the mutual influence coefficient between the risk factors. β_*ij*_ indicates the influence of the existence of indicator *i* to indicator *j*. That is to say, β_*ij*_ indicates the influence of the existence of indicator *i* to indicator *j*.


$$\begin{array}{l} \beta_{ij}=\\ \;\{\begin{array}{l} +\text{ The existence of indicator }i\text{ contributes to the development of indicator }j\\ -\text{ The presence of indicator }i\text{ inhibits the development of indicator }j \end{array} \end{array}$$

The absolute value of β_*ij*_ is taken according to the existence of indicator *i* to the impact of indicator *j* to take the value. If β_*ij*_ = 0, it means that the existence of indicator *i* to the indicator *j* has no impact. The evaluation of the mutual influence coefficient between the indicators constitutes a matrix, called the cross-reinforcement matrix. We will use the DEMATEL method of the mutual comprehensive impact of the options matrix to calculate the mutual influence matrix of the risk factors.

Step 3: calculate the weight vector *w* reflected by the mutual influence of the evaluated alternatives. It is called the influence weight. Calculate using the formula:


(8) $$\begin{array}{l} w_{l}=\frac{\sum_{j=1}^{k}\beta_{ij}w_{j}}{\sum_{i=1}^{k}\sum_{j=1}^{k}\beta_{ij}w_{j}}\left(k\;is\;the\;number\;of\;elements\;in\;the\;s\;layer\right) \end{array}$$

Step 4: combine the value weights and impact weights to calculate the new weights for each evaluation indicator *w*_*s*_.


(9) $$\begin{array}{l} w_{s}=\alpha w_{\text{j}}+\left(1-\alpha \right)w_{l},\;0\leq \alpha \leq 1 \end{array}$$

In general, α takes the value of 0.5:


(10) $$\begin{array}{l} w_{s}=\frac{1}{2}\left(w_{j}+\frac{\sum_{j=1}^{k}\beta_{\text{ij}}w_{j}}{\sum_{i=1}^{k}\sum_{j=1}^{k}\beta_{\text{ij}}w_{j}}\right) \end{array}$$

Step 5: The final weight ordering can be obtained as shown in Table 6 by calculating the above steps.

**Table 6:** Corrected weights and rankings.

**First-level indicators**	**Secondary indicators**	**Sum of cross-reinforcements**	**Initial weight**	**Affect the weight**	**Standardization**	**Rank**
Circumstances risk (CR) /0.2595	CR1	0.2990	0.0451	0.0393	0.0422	11
	CR2	0.1776	0.1436	0.0233	0.0835	3
	CR3	0.0636	0.0192	0.0084	0.0138	20
	CR4	0.2766	0.0448	0.0363	0.0406	12
	CR5	0.4918	0.0945	0.0646	0.0796	4
Language and cultural risk (LC)/0.2371	LC1	0.9848	0.0542	0.1294	0.0918	2
	LC2	0.2960	0.0075	0.0389	0.0232	19
	LC3	0.6024	0.0488	0.0792	0.0640	5
	LC4	0.3781	0.0199	0.0497	0.0348	16
	LC5	0.2652	0.0119	0.0348	0.0234	18
Academics risk (AR)/0.3756	AR1	0.4312	0.0347	0.0566	0.0457	8
	AR2	0.2626	0.0145	0.0345	0.0245	17
	AR3	0.6010	0.0373	0.0790	0.0581	6
	AR4	0.5398	0.0373	0.0709	0.0541	7
	AR5	0.3724	0.0373	0.0489	0.0431	9
	AR6	0.4798	0.1512	0.0630	0.1071	1
	AR7	0.1167	0.0707	0.0153	0.0430	10
Opportunity risk (OR)/0.1277	OR1	0.4637	0.0185	0.0609	0.0397	13
	OR2	0.0425	0.0098	0.0056	0.0077	21
	OR3	0.0039	0.0053	0.0005	0.0029	22
	OR4	0.3525	0.0293	0.0463	0.0378	15
	OR5	0.1098	0.0647	0.0144	0.0396	14

## 4 Discussion

### 4.1 Analysis of secondary indicators

To identify critical risk factors, we prioritize the top 23% (five out of 22 factors) based on cumulative weight contribution (45%), aligning with exploratory factor analysis conventions for retaining dominant components (Hair et al., 2010). This approach ensures focus on factors with the highest practical impact (Nouh et al., 2023). Therefore, the analysis focuses on the first five factors.

Compared to Lin et al. (2023) evaluated study risks in two countries by using FCE-AHP model with safety and education as important aspects, this study reveals self-management ability as the leading factor, highlighting the importance of individual agency in risk mitigation by a DEMATEL-integrated model. This discrepancy underscores the value of our hybrid approach in capturing latent factor interactions.

This study's hybrid model reveals that self-management ability (AR6) has a stronger total influence (direct + indirect effect = 0.234) due to its correlations with academic adaptation (AR2–AR7) and psychological pressure (OR3). Self-management ability (AR6) ranked highest and was considered the most important risk factor. People with good self-management ability (AR6) are more productive, which positively impacts their studies (Al-Abyadh and Abdel Azeem, 2022). Chinese students should pay attention to the driving effect of this factor and take full advantage of personal initiative to improve themselves during their time studying abroad.

Language ability (LC1) is the ability of Chinese students to use foreign languages to learn, analyze, and communicate. It is a combination of expressive, communicative, and analytical skills. It is a dominant evaluation index for international universities recruiting Chinese students. Students with good language ability (LC1) have a wider range of opportunities in the future. This ability is developed before they study abroad and will be needed and improved at any time during their studies abroad. Good language ability (LC1) makes it easier for students studying abroad to adapt to social life and academic circumstances in the host country. It plays a vital role in the smooth and successful completion of their studies. Language ability (LC1) will become a favorable tool for obtaining job and development opportunities after graduation (Bousmah et al., 2021; Marini et al., 2019). Therefore, it is important to consider the sufficient language ability (LC1) they have already obtained before departure and to improve it during their studies for Chinese students studying abroad.

The host country policy (CR2) is a significant indicator of a country's politics, economy, and social culture (Giovanis and Akdede, 2021). A friendly and relaxed international student policy can help international universities attract more Chinese students. However, policy changes, especially those concerning foreign students, may lead to the continuation or interruption of students studying abroad (Sina, 2021). Chinese students must pay close attention to changes and their impact on policies related to international students after experiencing SARS and COVID. This suggests the need to give sufficient attention to this factor.

The economic conditions of the host country (CR5) are crucial for the development of a country. They affect the country's development and indirectly influence all aspects of a student's situation. A strong economy improves the standard of living of the country and its people and enables international students to better engage in intercultural communication and dissemination. Host countries should focus on their economic conditions and create a favorable economic environment to attract more international students. Simultaneously, international students need to consider whether the economic situation of the country where they are studying aligns with their expected development and select a country that suits them.

Values (LC3) are the perceptions, understandings, judgments, or choices made based on certain ways of thinking. They represent a kind of thinking or value orientation that people use to determine things and decide what is right and wrong. Differences in values will expand cultural distance, induce cultural shock, and increase the difficulty of cross-cultural adaptation (Kim, 1977). The results suggest a measure for international universities to improve the study experience of Chinese students. It should be seen as a process of integrating multiple cultures and values in the admission, management, and teaching of international students. When facing cultural and value differences, it is necessary to respect the cultures and values of international students. They should learn to accept the beliefs of different countries, correctly handle the issue of multiple value conflicts, and not defame the culture of other countries.

### 4.2 Analysis of first-level indicators

The weights of the four level 1 indicators can be obtained from academic risk (AR), circumstantial risk (CR), language and cultural risk (LC), and opportunity risk (OR) in descending order of importance.

Academic risk (AR) is crucial for international students, affecting their current academic status and future life. International students need to be careful in the process of making academic choices. Circumstantial risk (CR) involves the uncertainty between countries located in a broader context, which can affect the adaptation of international students. This uncertainty may cause international students to be unable to adapt to various situations, such as differing policies, systems, and environmental factors, resulting in fundamental differences and difficulties in completing their studies. International students need to understand these conditions in advance and make good psychological preparations. Language and cultural risk (LC) will affect the daily lives of students. Poor communication and difficulties in cultural adaptation will lead to challenges in daily interactions, which can impact the physical and mental health of international students in the long run. Opportunity risk (OR) is also an aspect that international students need to pay attention to. Their physical and mental health and economic capacity during their studies can trigger a series of chain reactions, which should be emphasized by the host country.

## 5 Conclusions

### 5.1 Conclusions and countermeasures

We analyzed the key influences affecting international students using the DEMATEL method and the cross-reinforcement matrix method to improve AHP. Ultimately, five risk factors are considered the most important: self-management ability, language ability, host country policy, economic conditions of the host country, and values. Meanwhile, the primary indicator of academic risk (AR) needs to be given sufficient attention. These discussions provide a multi-perspective, prioritized view of risk control in choosing and making decisions about studying abroad. Countermeasures to address the risk factors during the study abroad process are as follows:

(1) International students should recognize the process, stages, and long-term nature of studying abroad. They should recognize the difficulties in language, specialty, psychology, culture, and safety in the process of cross-cultural learning. They should also improve their self-management abilities and actively cope with various difficulties encountered in their studies and lives abroad. Because international students often lack control over their daily lives, they need to improve their self-management skills. On the one hand, they should strengthen their self-control while living abroad. They can set achievable goals and specify goals at each stage to improve their time management skills and motivation to study. On the other hand, they should balance work and rest, ensuring they get enough rest through proper exercise, sufficient sleep, and other recreational activities. In addition, they should learn to remain committed after setting a goal, avoiding excessive delays in achieving it and engaging in deep self-reflection.
(2) They should clarify their intentions for studying abroad, fully consider their abilities and career plans, and identify any skills or qualities they have or lack, such as knowledge, economic status, language skills, interpersonal skills, and stress resistance. They should learn to use foreign languages for daily communication and plan further for their future lives. Language proficiency is an indispensable factor for studying abroad. Before studying abroad, international students should actively participate in various language tests, as these tests can fully and objectively reflect an individual's ability level. They serve as a critical assessment index for both interviewers and international students, relating directly to the future work and development of international students. Some international students often lose confidence in adapting to the host country because of insufficient language ability, affecting their academic and personal lives. Therefore, international students should clearly recognize their language proficiency and actively work to improve their language skills.
(3) Changes in national policies may significantly impact international students and their host countries. Each country should pay great attention to talent education issues, formulate strategies to adapt to the global talent market, improve the quality of education, ensure a competent faculty, and provide high-quality teaching resources. Additionally, they should expand international cooperation to improve international recognition. The interaction and communication between international students and local teachers and students should be strengthened to guide international students in effectively fulfilling their roles. Investment in language support, psychological counseling, and employment counseling should be increased. Furthermore, host countries should closely monitor the interests of international students to facilitate their gradual acceptance of foreign cultures in terms of cognition, psychology, and behavior.
(4) The economy of the host country and the degree of its economic development affect international students' perceptions of the country. The more economically developed a region is, the more attractive it becomes to international students. International students should consider the economic development of the country when deciding where to study abroad. If economic development of the host country is relatively backward, international students may experience more discomfort during their studies, negatively impacting their overall experience. Therefore, host countries should pay attention to domestic economic development to create a safer and more stable environment for international students. Moreover, the economic conditions of developed countries are more appealing to international students than those of countries with lower economic development.
(5) The values of international students will affect their study abroad experiences. Different geographic locations produce different values, which can lead to fundamental differences and discrepancies. Consequently, international students often need to integrate the differing values of various regions during their studies abroad. It is essential to respect the values of other international students during the integration process. They must learn to accept the beliefs of different countries in the face of value differences. While studying in a foreign country, they should learn to address multiple value conflicts independently and refrain from denigrating the culture of other countries in response to environmental changes.

This study successfully identified five key risk factors—self-management ability (AR6), language ability (LC1), host country policy (CR2), economic conditions (CR5), and values (LC3)—using a hybrid AHP–DEMATEL–cross-reinforcement matrix approach, addressing the research goal of systematic risk factor identification. The results validate the hypothesis that factor interdependencies significantly influence risk severity, providing a robust framework for risk prediction.

### 5.2 Limitations

We consider the complex relationships and the importance of various factors when analyzing the risk factors of Chinese students studying abroad. We use DEMATEL and a cross-reinforcement matrix to enhance the hierarchical approach and minimize the influence of individual subjective factors on the results. However, there are still limitations in the research process.

The methodology used in this paper lacks actual survey data to analyze from the perspective of individual international students. A variety of models, such as structural equation models or explanatory structural models, are needed to validate the pathways between the influencing factors and their impacts on international students in the future. The results would be more convincing.

Future research could expand the sample to include student surveys for empirical validation, apply machine learning to model dynamic risk changes, or compare risk profiles across host countries (e.g., English vs. non-English speaking nations). Additionally, integrating cost-benefit analysis into the framework would enhance its utility for study-abroad decision-making.

## Data Availability

The original contributions presented in the study are included in the article/supplementary material, further inquiries can be directed to the corresponding author.
